# *Erigeron annuus* Extract Alleviates Insulin Resistance via Regulating the Expression of Mitochondrial Damage and Endoplasmic Reticulum Stress-Related Genes

**DOI:** 10.3390/nu15122685

**Published:** 2023-06-09

**Authors:** Hyo Kyu Lee, Youn Hee Nam, Sung Woo Shin, Min Cheol Kim, Jung In An, Na Woo Kim, Ji Heon Shim, Sunitha Srinath, Bin Na Hong, Jong Hwan Kwak, Tong Ho Kang

**Affiliations:** 1 Department of Oriental Medicine Biotechnology, College of Life Sciences and Graduate School of Biotechnology, Kyung Hee University, Global Campus, Yongin 17104, Gyeonggi-do, Republic of Korea; 2 School of Pharmacy, Sungkyunkwan University, Suwon 16419, Gyeonggi-do, Republic of Korea

**Keywords:** *Erigeron annuus*, insulin resistance, zebrafish, mitochondrial damage, endoplasmic reticulum stress

## Abstract

Diabetes is a prevalent and debilitating metabolic disorder affecting a large population worldwide. The condition is characterized by insulin resistance and impaired function of pancreatic β-cells, leading to elevated blood glucose levels. In this study, the antidiabetic effects of *Erigeron annuus* extract (EAE) on zebrafish with damaged pancreatic islets caused by insulin resistance were investigated. The study utilized the zebrafish model to monitor live pancreatic islets. RNA sequencing was also conducted to determine the mechanism by which EAE exerts its antidiabetic effect. The results showed that EAE was effective in recovering reduced islets in excess insulin-induced zebrafish. The effective concentration at 50% (EC_50_) of EAE was determined to be 0.54 μg/mL, while the lethal concentration at 50% (LC_50_) was calculated as 202.5 μg/mL. RNA sequencing indicated that the mode of action of EAE is related to its ability to induce mitochondrial damage and suppress endoplasmic reticulum stress. The findings of this study demonstrate the efficacy and therapeutic potential of EAE in treating insulin resistance in zebrafish. The results suggest that EAE may offer a promising approach for the management of diabetes by reducing mitochondrial damage and suppressing endoplasmic reticulum stress. Further research is required to establish the clinical application of EAE in diabetic patients.

## 1. Introduction

*Erigeron annuus* (L.) Per. is a member of the Asteraceae family and is indigenous to North America. It is commonly known as daisy fleabane and has been introduced and extensively distributed in Korea, where it is used as a vegetable [1,2,3]. The plant has been utilized in traditional medicine for the treatment of dyspepsia, enteritis, hepatitis, and hematuria [4]. Current research has revealed that this plant is a rich source of various bioactive compounds, including flavanones, erigeron flavanones, sesquiterpenoids, ergosterol peroxide, caffeic acid, and pyromechanic acid, among others [5,6,7,8,9].

The aim of this study was to assess the potential antidiabetic properties of *E. annuus* in zebrafish with pancreatic islet damage arising from insulin resistance. Despite prior investigations demonstrating the anti-obesity and antioxidant capabilities of *E. annuus*, its potential as an agent for managing diabetes has yet to be explored.

Diabetes is a common metabolic disorder characterized by insulin resistance and impaired pancreatic β-cell function. Prolonged exposure to high levels of insulin has been shown to lead to insulin resistance and, eventually, type 2 diabetes (T2D) development. Insulin is produced by β cells located in the pancreatic islets [10,11]. However, over time, islet β-cell compensation for insulin resistance fails, resulting in a progressive loss of β-cell function [12]. While the correlation between β-cell function and β-cell mass may not always be direct, the apoptosis-induced loss of β-cell mass can serve as an indicative factor for the initiation of type 2 diabetes development [13]. The pathogenesis of insulin resistance is complex and multifactorial. Inflammation, endoplasmic reticulum stress, and mitochondrial-derived oxidative stress are key factors involved in the development of insulin resistance [14,15,16,17]. Chronic inflammation, which can result from obesity, plays a significant role in the development of insulin resistance [14]. Endoplasmic reticulum stress, which is triggered by excess lipid accumulation, can lead to the unfolded protein response and, ultimately, insulin resistance [15]. Similarly, mitochondrial-derived oxidative stress, which is caused by excess nutrient intake, can lead to insulin resistance [16,17].

In recent years, the zebrafish (*Danio rerio*) has emerged as an animal model for investigating the mechanisms underlying pathologies resulting from transformed metabolism, as evidenced by its increasing use in RNA sequencing studies [11,18,19,20]. Due to its high similarities in organ physiology and metabolism to mammals, the zebrafish has become an attractive model for studying diabetes and its related diseases [21,22,23,24]. This zebrafish model has been shown to exhibit insulin resistance and diabetes, making it suitable for evaluating antidiabetic candidates through the assessment of pancreatic islet recovery and gene expression profiles. Thus, the zebrafish model offers significant potential for drug testing and discovery in the context of insulin resistance and diabetes [21].

In this research, live pancreatic islets were monitored utilizing the excess insulin-induced zebrafish model. *E. annuus* extract (EAE) was evaluated for the potential of recovery of pancreatic islets, which were damaged due to insulin resistance in zebrafish. In order to confirm the composition of EAE, high-performance liquid chromatography (HPLC) was conducted. Furthermore, the recovery effect of the major components on the damaged pancreatic islets was also evaluated. RNA sequencing was performed to investigate the mechanism by which EAE exerts its anti-diabetic effect. The results of the study showed that EAE is related to the oxidative phosphorylation pathway and protein processing in the endoplasmic reticulum.

In conclusion, the study aimed to demonstrate the preventive and therapeutic effects of EAE on diabetes in zebrafish and to shed light on its potential modes of action.

## 2. Materials and Methods

### 2.1. Materials

Human recombinant insulin, pioglitazone, and sea salts were obtained from Sigma Chemical Co. (St. Louis, MO, USA). 2-(N-(7-nitrobenz-2-oxa-1,3-diazol-4-yl)amino)-2-deoxyglucose (2-NBDG) was obtained from Invitrogen (Waltham, MA, USA). Fluorescence microscopy analysis was performed using an Olympus IX70 microscope (Olympus, Tokyo, Japan), and image analysis was conducted using Focus Lite software (V 2.90, Focus Co, Suwon, Republic of Korea) and Image J software (V 1.50i, National Institutes of Health, Bethesda, MD, USA). The 70% ethanol extract of *Erigeron annuus* (EAE) was donated by the Nakdonggang National Institute of Biological Resources (Sangju, Republic of Korea).

### 2.2. HPLC Analysis of EAE

HPLC analysis of chlorogenic acid (CA) was based on a modification of a previous report [25,26,27,28]. To characterize *E. annuus* extract, quantitative analysis was conducted using a reversed-phase (RP) C_18_ HPLC. HPLC analysis was performed on a Knauer Smartline system consisting of a Manager 5000, Pump 1000 (×2), UV Detector 2500, and a Phenomenex Kinetex^®^ 5 μm C18 100 Å column (150 × 4.6 mm). The eluent consisted of acetonitrile (A) and 0.3% formic acid in water (B). The gradient profile was as follows: 0–10 min, isocratic elution with 1% A in B; 10–13 min, linear change from 1% to 15% A in B; 13–35 min, isocratic elution with 15% A in B; 35–45 min, linear change from 15% to 100% A in B. The column oven temperature, flow rate, and UV absorption were set at 30 °C, 1 mL/min, and 325 nm wavelength, respectively. Standard working solutions for chlorogenic acid were prepared by serial dilution with methanol to give concentrations of 500, 250, 100 and 50 μg/mL, respectively. The content of chlorogenic acid in the extract was determined using a regression equation for chlorogenic acid. In order to validate the HPLC method, various parameters were assessed for chlorogenic acid, including linearity, limit of detection (LOD), limit of quantification (LOQ), precision, and accuracy.

### 2.3. Zebrafish

The adult zebrafish were housed in a zebrafish S-type system (1500 [W] × 400 [D] × 2050 [H] mm) (Daejeon, Republic of Korea), under a controlled 14 h light:10 h dark cycle at a temperature of 28.5 °C. To obtain zebrafish larvae, two pairs of adult zebrafish were placed in a spawning box overnight, and spawning occurred during a 30 min period of light the next day. Zebrafish embryos were collected at 3 h post-fertilization, incubated, and maintained in a 0.03% sea salt solution, under a 14 h light:10 h dark photocycle, in an incubator set at 28.5 °C.

### 2.4. Ethics Statement

The present study adhered to standard zebrafish protocols and was conducted with the approval of the Animal Care and Use Committee of Kyung Hee University [KHUASP(SE)-15-10], in accordance with ethical guidelines for animal research. All experimental procedures involving zebrafish were carried out in compliance with these established protocols.

### 2.5. Efficacy of EAE and CA in Treating Excess Insulin-Induced Pancreatic Islet Damage in Zebrafish

Three-day post-fertilization (dpf3) wild-type zebrafish (*n* = 20) were experimentally studied to evaluate insulin treatment effects on pancreatic islets. The zebrafish larvae were placed into six-well plates and subjected to 0.1 μM pioglitazone, 1 μg/mL EAE or 0.1 μM CA for 24 h after a 48 h insulin treatment with a concentration of 10 μM. Pioglitazone, belonging to the thiazolidinedione class, acts as an agonist of peroxisome proliferator-activated receptors (PPARs), leading to an improvement in hyperglycemia, reduction in hyperinsulinemia, and enhancement of β-cell function in multiple insulin-resistant animal models [29,30]. In the present study, pioglitazone was utilized as a positive control. The pancreatic islets were subsequently visualized by staining the larvae with 40 μM 2-NBDG for 30 min and rinsing with a 0.03% sea salt solution for 20 min. To facilitate imaging, the zebrafish were anesthetized using tricaine and mounted on their sides with only the left eye visible. Fluorescence microscopy was used to capture images of the pancreatic islets, which were analyzed using the Focus Lite (V 2.90) and Image J software (V 1.50i).

Group 1: Control group (CON). Only treatment with 0.03% sea salt solution. *n* = 20.
Group 2: Insulin-treated group (INS). Treatment with 0.03% sea salt solution after 10 μM insulin treatment for 48 h. *n* = 20.
Group 3: Pioglitazone-treated group (PIO). Treatment with 0.1 μM pioglitazone for 24 h after 10 μM insulin treatment for 48 h. *n* = 20.
Group 4: *Erigeron annuus* extract-treated group (EAE). Treatment with 1 μg/mL EAE for 24 h after 10 μM insulin treatment for 48 h. *n* = 20.
Group 5: Chlorogenic acid-treated group (CA). Treatment with 0.1 μM CA for 24 h after 10 μM insulin treatment for 48 h. *n* = 20.

### 2.6. The 50% Effective Concentration (EC_50_) of EAE

The present study evaluated the efficacy of various concentrations of EAE, ranging from 0.01 μg/mL to 10 μg/mL, in zebrafish. The median effective concentration (EC_50_) was determined by means of non-linear regression analysis, employing GraphPad Prism version 5.01 software, developed by GraphPad Software (San Diego, CA, USA).

### 2.7. The 50% Lethal Concentration (LC_50_) Values of EAE

Zebrafish were subjected to eight distinct concentrations (10, 50, 100, 200, 400, and 500 μg/mL) of EAE, and the determination of LC_50_ values was performed through non-linear regression, utilizing GraphPad Prism version 5.01 software.

### 2.8. Therapeutic Index (TI)

The therapeutic index (TI) is a crucial parameter used to assess the safety profile of a drug for a specific treatment. The TI is also known as the therapeutic window or safety margin, and it is defined as the ratio of the toxic concentration (LC_50_) to the therapeutic concentration (EC_50_) of the drug. The calculation of TI is performed using the following equation:

TI = LC_50_/EC_50_

In essence, the TI provides a quantitative measure of the balance between the desired therapeutic effects of a drug and its potential toxic effects. Understanding the TI is critical for optimizing dosing regimens and minimizing the risk of adverse events associated with drug treatment.

### 2.9. mRNA Sequencing and Pathway Analysis

Three-day post-fertilization (dpf3) zebrafish larvae were maintained in six-well plates and subjected to treatment with 10 μM insulin for 48 h. After the treatment, the zebrafish larvae were thoroughly rinsed with 0.03% sea salt solution for another 48 h, and finally washed with phosphate-buffered saline (PBS). Total RNA was extracted from the treated zebrafish larvae using the Trizol RNA Isolation Reagent (Invitrogen, Carlsbad, CA, USA), followed by further purification using the RNeasy Mini Kit (QIAGEN, Hilden, Germany) to eliminate genomic DNA.

The quantity and quality of the total RNA was evaluated using the Agilent 2100 Bioanalyzer (Agilent, Santa Clara, CA, USA). RNA sequencing libraries were prepared from the purified total RNA using the TruSeq Stranded mRNA Sample Preparation Kit (Illumina, San Diego, CA, USA). The quality and size of the entire library was then assessed using the Agilent 2100 Bioanalyzer. The libraries were quantified by quantitative polymerase chain reaction (qPCR) using a CFX96 real-time system (Bio-Rad, Hercules, CA, USA) and sequenced on a NextSeq500 sequencer (Agilent) with paired-end 75 bp and single 8 bp index read runs. The RNA analysis raw data were converted into sequence data and stored as FASTQ files.

Differentially expressed genes (DEGs) were determined based on an absolute fold change (FC) greater than 1.4 and a *p*-value less than 0.05. The differentially expressed gene data sets were processed using the Kyoto Encyclopedia of Genes and Genomes (KEGG) expression database. Further analysis of the differentially expressed genes was performed using EnrichR for Wiki Pathways analysis [22].

### 2.10. RT-PCR

The extraction of total RNA was performed, and cDNA synthesis was carried out using the Rever Aid First Strand cDNA Synthesis Kit (Thermo Fisher Scientific Korea Ltd., Seoul, Republic of Korea) following the manufacturer’s protocol. The primer sequences used for gene expression analysis are detailed in Table 1. Real-time polymerase chain reaction (RT-PCR) was conducted using SYBR Green Master Mix (Applied Biosystems, Thermo Fisher Scientific Korea Ltd., Seoul, Republic of Korea). The RT-PCR conditions are specified in Table 2. β-actin was employed as the internal control gene, and the relative gene expression levels were calculated using the −2^△△Ct^ method [23].

### 2.11. Statistical Analysis

Statistical analyses were performed using GraphPad Prism version 5. The data were reported as the mean ± standard error of the mean (SD). The statistical significance of the results was assessed using a repeated one-way analysis of variance (ANOVA), followed by Tukey’s post hoc test. The criterion for statistical significance was set at a probability level of *p* < 0.05.

## 3. Results

### 3.1. Phytochemcial Characterization of EAE

**Figure 1:**
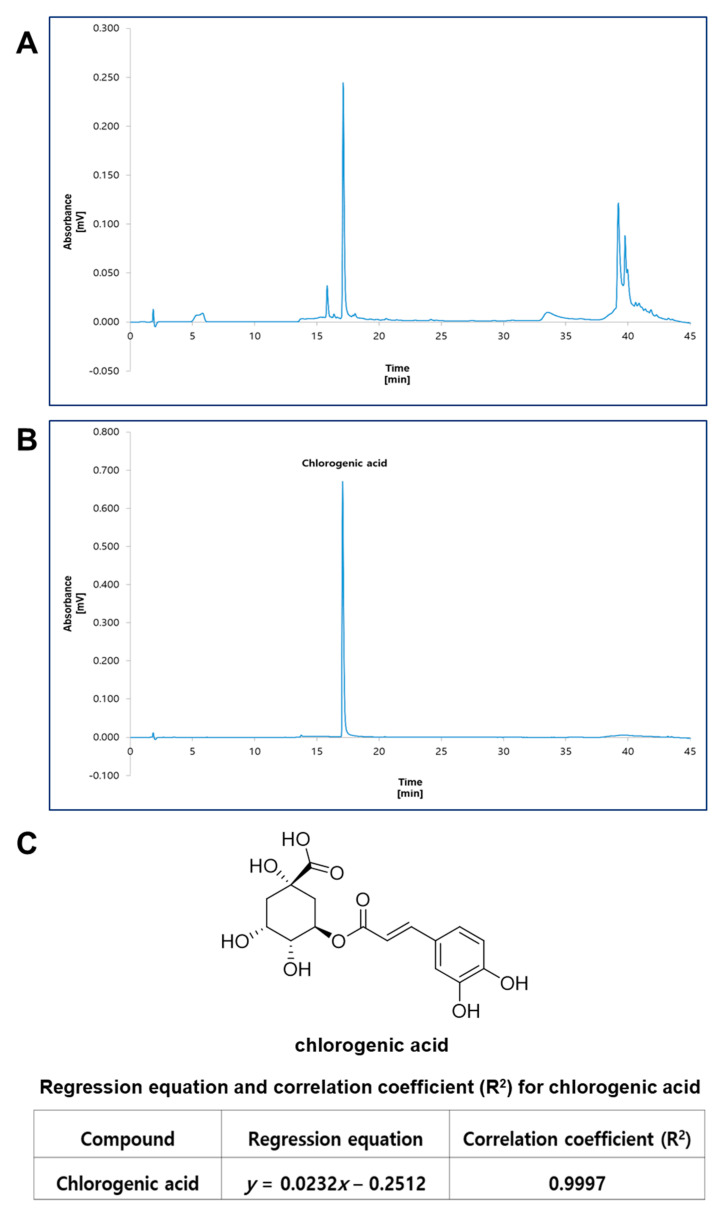
High-performance liquid chromatography (HPLC) analysis for EAE. (**A**) HPLC-UV chromatogram of EAE. (**B**) HPLC-UV chromatogram of chlorogenic acid as a standard compound. The major component in the extract was identified and quantified by using HPLC analysis. HPLC analysis was carried out via gradient elution on a Phenomenex Kinetex C18 column (150 × 4.6 mm, 5 µm). The flow rate, column oven temperature, and UV wavelength for detection were set at 1 mL/min, 30 °C, and 325 nm, respectively. (**C**) Chemical structure of chlorogenic acid, and regression equation and correlation coefficient for the standard compound.

As shown in the HPLC chromatogram of *E. annuus* extract (Figure 1A), there is a major peak identified as chlorogenic acid by comparison with the standard material (Figure 1B). The content of chlorogenic acid in the extract was determined as 16.10 μg/mg extract by using the regression equation (Figure 1C). The linearity of the method was determined by a regression equation (*y =* 0.0232*x* − 0.2512). A high degree of linearity (R^2^ = 0.9997) was observed, indicating a strong correlation between the peak areas and chlorogenic acid concentrations. The sensitivity of the HPLC method was evaluated by determining the limit of detection (LOD) and limit of quantification (LOQ). The LOD was found to be 0.93 µg/mL, and the LOQ was 3.11 µg/mL (Table 3). Precision was assessed to determine the repeatability and reproducibility of the method. The relative standard deviation (RSD) of the peak areas was calculated, and the method demonstrated excellent intra-day precision, with an RSD of 1.77%, and inter-day precision, with an RSD of 1.99% (Table 4). Accuracy was evaluated by spiking known amounts of chlorogenic acid into a sample matrix and comparing the measured concentrations to the expected values. The HPLC method demonstrated high accuracy, with recovery rates of 98.14%, and an RSD of 0.54% (Table 5).

### 3.2. Efficacy of EAE and CA on Insulin-Treated Zebrafish Larvae

**Figure 2:**
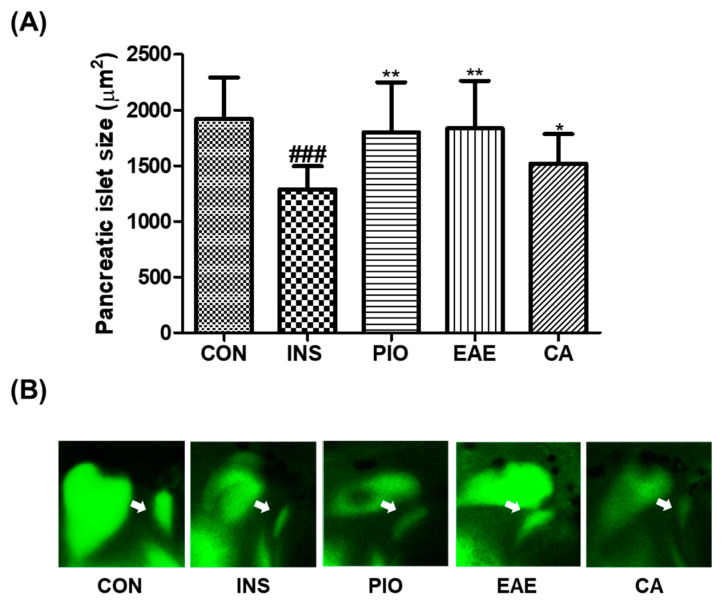
Effect of EAE and CA on insulin-treated pancreatic islet-damaged zebrafish. (**A**) Pancreatic islet size of each group. (**B**) Fluorescent images of pancreatic islet. (^###^ *p* < 0.001; compared to control), (* *p* < 0.05, ** *p* < 0.01; compared to INS). White arrows indicate the pancreatic islets. Control group (CON); insulin-treated group (INS); pioglitazone-treated group (PIO); EAE-treated group (EAE); chlorogenic acid-treated group (CA).

The average size of pancreatic islets in 7-day post-fertilization zebrafish was determined to be approximately 1916.9 ± 374.2 μm^2^. The treatment with insulin resulted in a significant decrease in the size of pancreatic islets (32.8%, *p* < 0.001) compared to the control group. On the other hand, treatment with pioglitazone (PIO) significantly increased the pancreatic islet size by 39.6% (*p* = 0.004). The EAE treatment group also showed a significant increase in pancreatic islet size (42.5%, *p* = 0.002). Although less recovered than the EAE treatment group, the CA treatment group also show a significant increase in pancreatic islet size (17.9%, *p* = 0.044), as depicted in Figure 2.

### 3.3. EC_50_ Values of EAE

**Figure 3:**
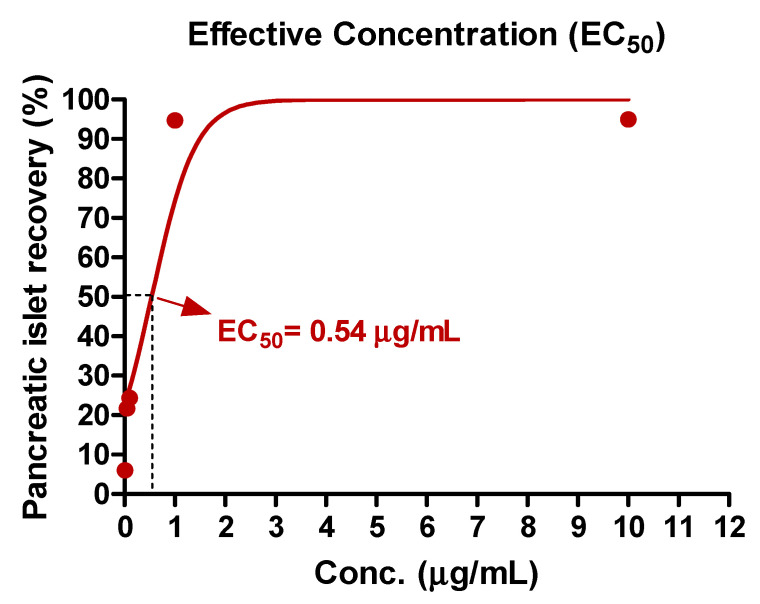
Concentration–effect curves of EAE. The EC_50_ of EAE was 0.54 μg/mL. The values are expressed as percentages of the baseline and each point was assessed in 0.01–10 μg/mL concentration range for determination of EC_50_.

A concentration–effect curve was generated using zebrafish treated with EAE at five different concentrations in order to determine the EC_50_ value. The calculated EC_50_ of EAE was found to be 0.54 μg/mL, as illustrated in Figure 3.

### 3.4. LC_50_ Values of EAE

**Figure 4:**
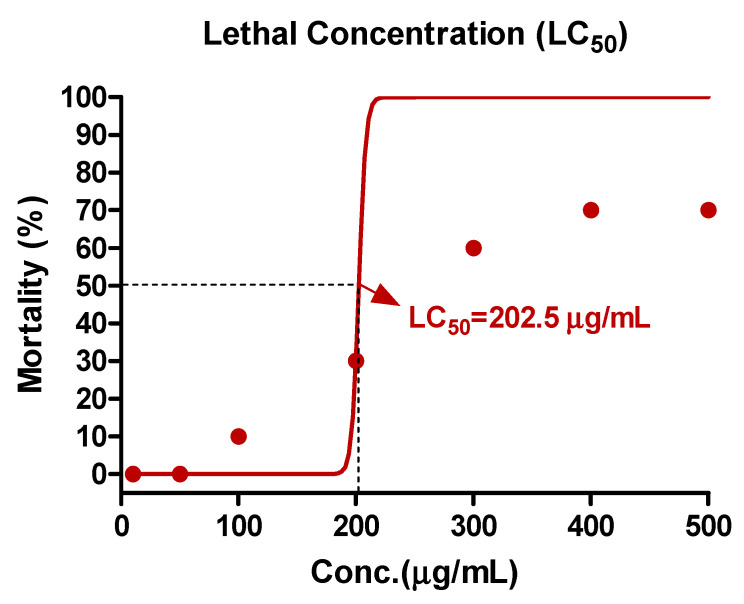
LC_50_ of zebrafish embryos exposed to EAE for 72 h. The LC_50_ of EAE was 202.5 μg/mL. The values are expressed as percentages of the baseline and each point was assessed in 10–500 μg/mL concentration range for determination of LC_50_.

In this study, the mortality of zebrafish exposed to EAE was analyzed to determine the lethal concentration (LC_50_). Zebrafish were exposed to seven different concentrations of EAE. The results indicated that the LC_50_ of EAE was determined to be 202.5 μg/mL, as presented in Figure 4.

### 3.5. Therapeutic Index (TI)

**Figure 5:**
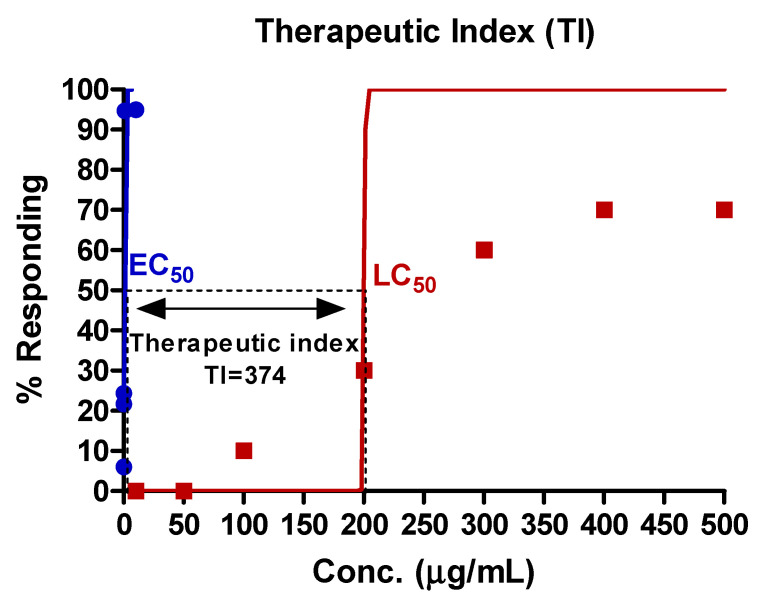
The therapeutic index (TI) of EAE. The TI of EAE was 374. Two non-linear curves for determining the EC_50_ and LC_50_ were expressed as blue and red line, respectively.

The therapeutic index (TI) of EAE was determined by the calculation of LC_50_/EC_50_. This index is widely recognized as an indicator of extract safety, with a higher TI implying a safer drug profile. Our results revealed that the TI of EAE was calculated to be 374 (as demonstrated in Figure 5).

### 3.6. The Quality Control of Sequencing Data

**Figure 6:**
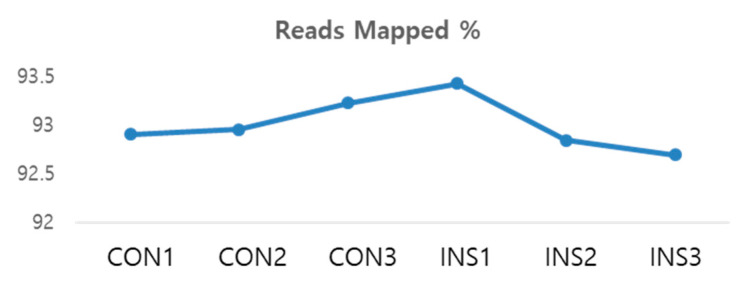
Average mapping rate.

Quality control of sequencing data is critical for ensuring accurate representation of gene expression products. First, we evaluated the sequencing and mapping quality of the data generated from a sample. Our analysis involved the use of Table 6 for sequencing information, which showed an average of 96.187% clean paired reads, and Figure 6 for mapping information, which indicated an average mapping rate of 93.013%. These results suggest that the sequencing quantity of the sample is sufficient to represent the real gene expression products, and the sequencing and mapping quality is adequate.

### 3.7. Differentially Expressed Genes by EAE in Insulin-Treated Zebrafish

**Figure 7:**
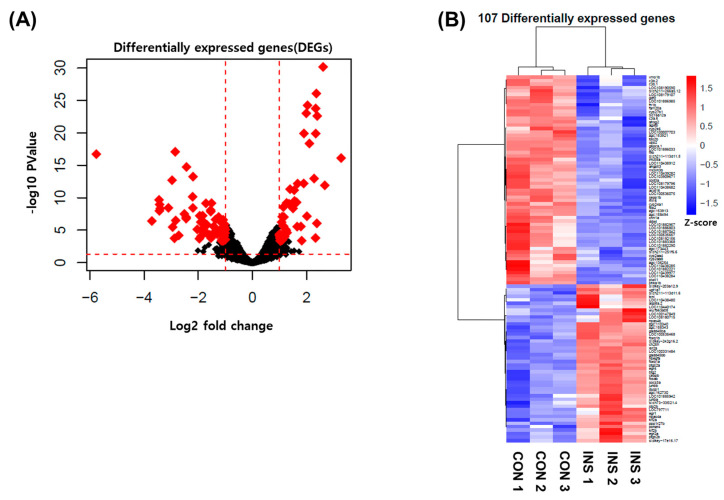
Differentially expressed genes (DEGs) by EAE in insulin-treated zebrafish. (**A**) Volcano plot of control + EAE vs. insulin resistance + EAE group. Of the total genes, 107 were significantly altered by insulin resistance + EAE (FDR < 0.05, |FC| > 1.0). (**B**) Heat map based on RNA-seq analysis of gene expression in EAE-treated zebrafish.

The mechanism of EAE on insulin resistance was investigated through transcriptome analysis to identify affected genes. RNA sequencing was utilized to examine differentially expressed genes (DEGs) in control or excess insulin-induced zebrafish treated with EAE. Out of the 19,422 genes expressed in EAE-treated zebrafish, 107 genes showed significant alterations (FDR < 0.05, |FC| > 2.0) in expression due to insulin resistance, as indicated by a volcano plot (Figure 7A). Among these 107 genes, 46 were upregulated and 61 were downregulated by insulin resistance, as depicted in Figure 7B.

**Figure 8:**
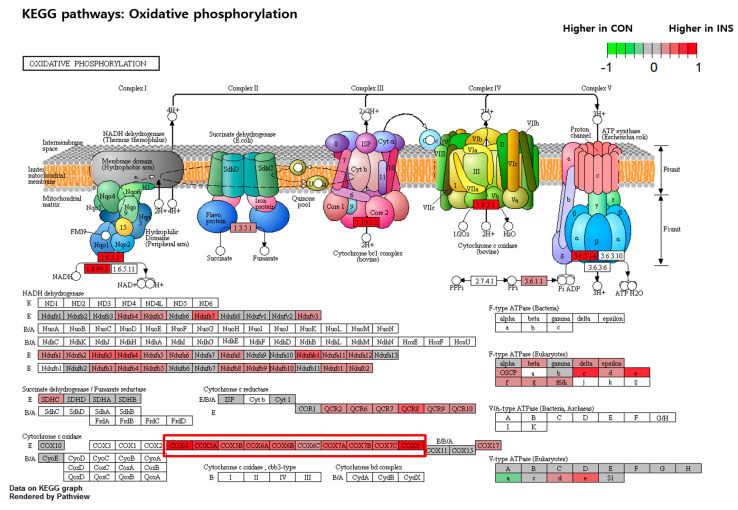
KEGG pathway analysis of the differentially expressed genes (DEGs) involved in oxidative phosphorylation. Green boxes indicate the DEGs by EAE treatment in the control group, and the red boxes show the DEGs by EAE treatment in the insulin-treated group. The DEGs highlighted by red rectangular mark in the figure represent significantly downregulated genes in EAE treatment in insulin-treated zebrafish.

**Figure 9:**
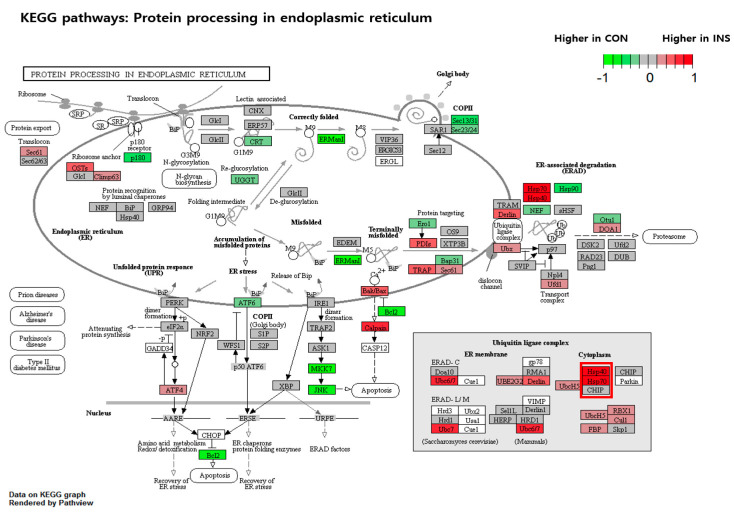
KEGG pathway analysis of the DEGs involved in protein processing in the endoplasmic reticulum. Green boxes indicate the DEGs by EAE treatment in the control group, and the red boxes show the DEGs by EAE treatment in the insulin-treated group. The DEGs highlighted by red rectangular mark in the figure represent significantly downregulated genes in EAE treatment in insulin-treated zebrafish.

Next, a KEGG analysis was conducted to determine the functional pathways of the differentially expressed genes in response to insulin resistance in the organism code ‘Danio rerio (zebrafish) [dre]’. The analysis revealed significant enrichment of pathways of oxidative phosphorylation (Entry: dre00190, Figure 8) and protein processing in the endoplasmic reticulum (Entry: dre04141, Figure 9) emerging as prominently affected pathways. The colors within the pathway diagrams corresponded to differentially expressed genes, highlighting their altered expression levels. These findings suggest that insulin resistance exerts a significant impact on metabolic processes, particularly in the areas of mitochondrial function and endoplasmic reticulum (ER) stress. Among the various differentially expressed genes within this pathway, we observed a particularly prominent downregulation of *COX4* and *HSP70* genes, along with *COX8* and *HSP40*, indicating their significant decrease in expression levels.

### 3.8. Changes in Gene Expression by EAE in Insulin-Treated Zebrafish

**Figure 10:**
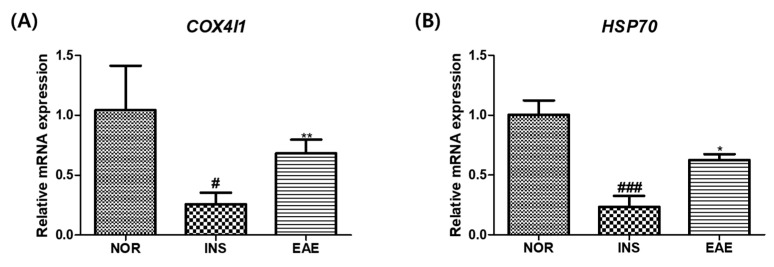
Quantitative RT-PCR using *COX4I1* and *HSP70* genes. Data are presented as means ± SD. (**A**) The *COX4I1* gene was determined by RT-PCR. (#) *p* < 0.05; compared to normal group (NOR). (**) *p* < 0.01; compared to insulin-treated group (INS). (**B**) The *HSP70* gene was determined by RT-PCR. (###) *p* < 0.001; compared to normal group (NOR). (*) *p* < 0.05; compared to insulin-treated group (INS).

The gene expression profiles of insulin-treated zebrafish were compared to those of the control group. Specifically, we compared the gene expression profiles of insulin-treated zebrafish to those of the control group and assessed the effect of EAE treatment on the expression levels of the *COX4I1* and *HSP70* genes. Our results indicated a significant decrease in the expression of both *COX4I1* and *HSP70* genes in the insulin-treated group, compared to the control group. However, administration of EAE resulted in a significant upregulation of both genes, as demonstrated in Figure 10 (*COX4I1*: *p <* 0.01; *HSP70*: *p <* 0.05). These findings suggest that EAE may have therapeutic potential for restoring gene expression levels in insulin-treated zebrafish.

## 4. Discussion

In this study, we aimed to assess the potential antidiabetic effects of EAE. The investigation found that EAE contains a compound called chlorogenic acid (CA), which has been shown to have antidiabetic effects [31,32,33]. Specifically, the concentration of CA in EAE was found to be 16.10 μg/mg.

Chlorogenic acid (CA), a naturally occurring phenolic compound, has been demonstrated to exert hypoglycemic effects in diabetic patients [34], as well as in animal models, including mice fed a high-fat diet [35] and diabetic zebrafish [36]. Notably, CA supplementation was found to significantly reduce fasting blood glucose levels in diabetic patients [34], while also exhibiting a marked glucose-lowering effect in high-fat-diet-fed mice [35]. Additionally, in diabetic zebrafish, CA was shown to significantly restore the function of damaged pancreatic islets [36]. We also confirmed the protective effect of CA in excess insulin-treated zebrafish model by recovering the size of damaged pancreatic islets.

This study utilized the zebrafish model to induce insulin resistance and reduction in islet mass via excessive insulin-induced damage to pancreatic islets. Consistent with prior work, this approach led to a reduction in pancreatic islet and β-cell size in the zebrafish [21]. To establish the diabetic zebrafish model, zebrafish were exposed to 10 μM of insulin for 48 h. Pancreatic islets were identified using 2-NBDG staining, which has the advantage of enabling visualization of the pancreas without the need for specialized equipment or technical expertise. Our observations revealed that excess insulin exposure led to significant damage in pancreatic islets and β-cells, and subsequent administration of EAE led to a significant recovery effect in zebrafish subjected to excessive insulin-induced pancreatic islet damage. When comparing the recovery effect of the EAE treatment group and the CA treatment group in the excess insulin-treated zebrafish, the EAE treatment was more effective, suggesting that the synergy of various components in the EAE has an anti-diabetic effect compared to only CA treatment.

The effective concentration at 50% (EC_50_) of EAE was determined to be 0.54 μg/mL, while the lethal concentration at 50% (LC_50_) was calculated as 202.5 μg/mL. The therapeutic index (TI), which is defined as the ratio of LC_50_ to EC_50_, was utilized to quantitatively compare the safety of EAE with other drugs. A larger TI value indicates a safer drug, whereas a smaller TI value suggests that caution should be taken in administering the drug and close monitoring of patients for potential toxicity [37]. In conclusion, our findings demonstrate that EAE is a safe drug based on its TI values.

In the current study, we conducted RNA sequencing (RNA-seq) to explore the mechanism underlying EAE. The precise cause of insulin resistance remains unclear, but several key mechanisms have been suggested, including oxidative stress, inflammation, mutations in insulin receptors, endoplasmic reticulum stress, and mitochondrial dysfunction [38]. The analysis of genes upregulated by insulin resistance revealed enrichment in pathways related to oxidative phosphorylation and protein processing in the endoplasmic reticulum. The oxidative phosphorylation pathway is involved in a broad range of cellular processes, including oxidative stress and impaired mitochondrial function [38]. Its attenuation is a well-established approach for treating insulin resistance and diabetes. Our results showed changes in the expression of *Cox* genes related to mitochondrial function, with particularly large changes in the expression of the *cox4* gene [39]. *Cox* genes, or cytochrome c oxidase genes, are crucial for the proper function of the electron transport chain (ETC) in the mitochondria, which produces ATP, the cell’s energy currency. The cytochrome c oxidase complex is the last enzyme complex in the ETC and plays a critical role in transferring electrons to oxygen to produce ATP. The *Cox* genes encode for different subunits of the cytochrome c oxidase complex, which are essential for its assembly and stability.

One of the *Cox* genes, *cox4*, codes for a protein subunit of the cytochrome c oxidase complex. This nuclear-encoded gene is crucial for the proper function of the COX complex, as it stabilizes the holoenzyme and facilitates the transfer of electrons from cytochrome c to molecular oxygen. Mutations in the *cox4* gene can cause various mitochondrial disorders and respiratory chain deficiencies, leading to severe health problems, such as neurodegeneration, muscle weakness, and developmental delays.

Recent studies have linked mutations in the *cox4* gene to impaired mitochondrial function and oxidative phosphorylation, which can contribute to insulin resistance and type 2 diabetes. Deficiencies in the COX complex due to mutations in the *cox4* gene can lead to various mitochondrial disorders, such as Leigh syndrome and cytochrome c oxidase deficiency, which can have significant health implications [40]. Reduced expression of *COX4I1* has been linked to decreased glucose uptake and insulin signaling, as indicated by a reduction in *GLUT4* expression, which may be partially dependent on *PPAR* expression. Additionally, *COX4I1* downregulation in obesity decreases protection against mitochondrial oxidative stress and increases metabolism, which may eventually result in type 2 diabetes [39].

The endoplasmic reticulum (ER) is an organelle in eukaryotic cells responsible for protein folding and processing. During times of metabolic stress, such as during obesity, high levels of glucose and fatty acids can lead to ER stress and the activation of the unfolded protein response (UPR). The UPR is an adaptive mechanism designed to reduce ER stress by decreasing protein synthesis and increasing the degradation of misfolded proteins. However, chronic ER stress, as seen in insulin resistance, can result in dysregulation of the UPR and contribute to the development of insulin resistance [41]. The findings of the study revealed a significant correlation between insulin resistance and alterations in metabolic processes, with a notable effect on mitochondrial function and ER stress. These results are consistent with previous research that has established a connection between insulin resistance and mitochondrial dysfunction, leading to oxidative stress and impaired insulin signaling [24,42,43,44]. Additionally, previous studies have demonstrated that ER stress in beta cells can disrupt insulin synthesis and sensitivity, further supporting the findings of this study [45].

The ER pathway is of significant importance in the context of diabetes, as protein processing within this pathway is closely tied to ER stress [46]. Numerous studies have explored this relationship, revealing that expression of the *HSP70* gene, which is involved in protein processing in the ER, is associated with insulin resistance. *HSP70* has a crucial role in the development of insulin resistance, as it contributes to inflammation, affects the mitochondrial function, and exacerbates ER stress [47]. A decrease in *HSP70* concentration leads to further activation of *JNK*, which in turn results in an increase in inflammation. Moreover, *HSP70* depletion has been shown to reduce mitophagy and mitochondrial biogenesis, thereby inhibiting fatty acid oxidation within the mitochondria. Furthermore, ER stress can activate *SREBP-1*, a transcription factor responsible for lipid gene expression [48]. Conversely, increased expression of *HSP70* has been shown to improve insulin sensitivity, as demonstrated in insulin-resistant zebrafish. The increased sensitivity was found to be linked to the pancreatic islet function of *HSP70* [49]. These findings highlight the complex and intertwined relationship between *HSP70*, ER stress, and insulin resistance in diabetes.

## 5. Conclusions

In conclusion, our study provides evidence of the efficacy and therapeutic potential of EAE in mitigating insulin resistance in zebrafish. We propose that the mechanism underlying EAE’s therapeutic effect may involve inducing mitochondrial damage and inhibiting endoplasmic reticulum stress. These findings shed light on the potential of EAE as a therapeutic agent for insulin resistance and offer insights into the molecular pathways involved in its mode of action. Further investigations are warranted to validate these observations and explore the translational potential of EAE in treating insulin resistance in human patients.

## Figures and Tables

**Table 1:** Primer sequences for RT-PCR.

Gene	Primer	Sequence (5′ to 3′)	NCBI Sequence
*COX4I1*	Forward	CGTCTTGTTGGTAAACGG	NM_214701.1
	Reverse	GGTAACAGGTGGACAAAC	
*HSP70*	Forward	GTCCTGGTGAAGATGAAGG	NM_001113589.1
	Reverse	CTCCACAGGATCTAGTGTTC	
	Reverse	GAAGGAAGACGTGTAGGTG	
	Reverse	GATCCTCTCCAGTTTCCTC	
*β-actin*	Forward	CGAGCAGGAGATGGGAACC	NM_131031.2
	Reverse	CAACGGAAACGCTCATTGC	

**Table 2:** Conditions of RT-PCR.

Step	Temperature	Time	Number of Cycles
Initialization	95 °C	10 min	1
Denaturation	95 °C	10 s	40
Annealing	60 °C	60 s	
Extension	72 °C	20 s	
Final extension	72 °C	60 s	1
Hold	4 °C	Hold	

**Table 3:** Retention time, correlation coefficient (R^2^), limit of detection (LOD), and limit of quantification (LOQ) for chlorogenic acid.

Compound	Retention Time (min)	R^2^	LOD (μg/mL)	LOQ (μg/mL)
Chlorogenic acid	17.2	0.9997	0.93	3.11

**Table 4:** Intra-day and inter-day precision and accuracy for chlorogenic acid.

Compound	Concentration (μg/mL)	Intra-Day (*n* = 3)		Inter-Day (*n* = 3)	
		RSD (%)	Accuracy (%)	RSD (%)	Accuracy (%)
Chlorogenic acid	50	1.77	98.09	1.99	98.75

**Table 5:** Recovery data for chlorogenic acid (*n* = 6).

Compound	Spiking Amount (μg/mL)	Recovery (%)	RSD (%)
Chlorogenic acid	50	98.14	0.54

**Table 6:** Sequencing information.

	Raw Read Counts	Q30 (%)	Clean Read Counts	Clean Read (%)
CON 1	80,681,844	96.24	77,675,166	96.27
CON 2	73,488,336	96.06	70,602,542	96.07
CON 3	117,477,450	94.94	113,192,176	96.35
INS 1	66,490,238	96.23	64,048,234	96.32
INS 2	88,797,948	96.55	85,268,344	96.02
INS 3	81,023,866	95.97	77,837,848	96.06

## Data Availability

Data are contained within the article.
